# Conductance Current and Space Charge Characteristics of SiO_2_/MMT/LDPE Micro-Nano Composites

**DOI:** 10.3390/ma13184119

**Published:** 2020-09-16

**Authors:** Hongtao Jiang, Xiaohong Zhang, Junguo Gao, Ning Guo

**Affiliations:** Key Laboratory of Engineering Dielectrics and Its Application, Ministry of Education, Harbin University of Science and Technology, Harbin 150080, China; jianghongtao012@163.com (H.J.); gaojunguo@hrbust.edu.cn (J.G.); guoninghust@163.com (N.G.)

**Keywords:** micro- and nanoparticles, adding order, crystallization, conduction current, space charge

## Abstract

Low-density polyethylene (LDPE) is one of the most comprehensive products used as insulation materials in power equipment. How to improve its dielectric properties by doping inorganic particles in LDPE has always been the focus of many researchers. In this paper, silica (SiO_2_) particles and montmorillonite (MMT) particles were added to LDPE, the order of adding particles was changed, and different micro-nano composites was made. The crystallization characteristics of composites were analyzed, the curves of the conductance current with the change of field intensity were analyzed, and the space charge distribution of each material were investigated. The results of crystallization show that the crystalline properties and crystallinity of the composites are higher than the matrix LDPE, the addition of SiO_2_ particles increases the composites’ crystallinity significantly, and the intercellular spacing of micro-nano composites is the smallest among all materials. The curve of conductance current versus electric field intensity shows that the tightness of the crystal structure can effectively hinder the movement of the molecular chain, inhibit carrier migration, while shortening the free travel of electrons, thereby reducing the electric conduction current of the material. The experimental results of the space charge accumulation curve further show that the compact crystal structure of the material is beneficial to the dissipation of space charge in the dielectric.

## 1. Introduction

High-voltage DC cable provides the possibility of accurate and real-time control of power transmission in a power system. At the same time, it can make the asynchronous interconnection of power grid safe and stable in different frequencies and incompatible states. It is still widely regarded at home and abroad as a cutting-edge technology. Low density polyethylene (LDPE) is widely used in the production of high voltage DC cable because of its excellent electrical insulation performance and processing characteristics [1,2]. However, in DC electric field, pure polyethylene materials still face many problems, the most serious is space charge accumulation. The accumulation of space charge easily leads to the distortion of a local electric field in the material that leads to the aging phenomenon of the material, which greatly shortens the life of the material and causes serious engineering loss [3]. Therefore, the key research focus of high-voltage power cables is to explore and tackle the technology to improve the performance of polyethylene [4,5,6,7].

In recent years, it has been found that the addition of inorganic particles in composite materials can effectively improve the breakdown field strength [8,9,10], suppress space charge [11,12,13,14,15,16,17,18,19] and regulate carrier flow [20,21,22]. Praeger et al. used propyl, octyl and octadecyl groups to treat the surface of SiO_2_ and studied the effect of functional group chain length on polyethylene/silica nanocomposites. They found that the chain length of the functional group will affect the motion mechanics process of charge transport, and at the same time, the removal of hydroxyl on the surface of SiO_2_ will make it hydrophobic, thus changing the charge transport behavior [23]. Panaitescu et al. doped nano-SiO_2_ particles and nano-Al_2_O_3_ particles in matrix LDPE by the melting-blending method and found that the concentration and type of nanoparticles have a significant impact on the mechanical and electrical properties of materials. They found that when the content of nanofiller was low (especially at 2%), the dispersion of nanopowder was better, the interfacial adhesion was enhanced, and the mechanical properties were the best. Among all of the composites, PE/2% nano-Al_2_O_3_ had better mechanical and dielectric properties [24]. Tjong et al. melted and blended 200 nm ZnO and 2 μm ZnO into LDPE. By the percolation theory, they explained the reason why the resistivity of the composite material dropped sharply when the content of ZnO was higher than 18 vol% and located the critical distance between particles in the two composites at 400 nm. When the distance between particles was lower than 400 nm, conduction occurs between ZnO particles via tunneling, resulting low resistivity [25].

Nanoparticles are easy to agglomerate, and it is difficult to achieve excellent dispersion in the composite system; this varies the conclusions drawn by different researchers. In order to improve the dispersion of nanoparticles in a polymer, people try to add micro- and nanoparticles into the matrix polymer at the same time, hoping to obtain composite materials with excellent performance through the interaction of micro- and nanoparticles. At present, existing studies have shown that adding micron particles together with nanoparticles into the matrix can not only avoid the agglomeration of nanoparticles, but also increase the performance of the matrix [26,27,28]. Montmorillonite (MMT) has a wide range of applications. After the modification process of experts and scholars, montmorillonite has been endowed with richer and unique properties, and has the reputation of “universal material”. Combining MMT with polymer will improve the mechanical, thermal and electrical properties of materials. Chi et al. prepared MMT/LDPE composite by melting and blending MMT with LDPE together. and tested the partial discharge resistance of the composite. They found that the uniform distribution and unique layered structure of MMT can effectively hinder the growth of electrical trees [29].

The nano-SiO_2_ and layered micro-MMT were doped into polyethylene matrix, both of which SiO_2_ and MMT doping mass fraction were 1 wt%. The MMT/LDPE, SiO_2_/LDPE, SiO_2_-MMT/LDPE and MMT-SiO_2_/LDPE micro-nano composites were prepared by melt blending, of which the order of adding micro- and nanoparticles is changed in SiO_2_-MMT/LDPE and MMT-SiO_2_/LDPE micro-nano composites. From the polarizing microscopic test and microcosmic test, the microcrystalline structure of each material could be explored. The composite conductivity current and space charge characteristics under different electric field intensity and order of adding micro-nano particles were discussed in this article, which could explore the impact mechanism of micro- and nanoparticles on internal electrical charges transport and space charge suppression property of composite In this article, a bridge between the microstructure and the macroscopic electrical properties is established, which provides a theoretical reference for understanding the influence of the microstructure on the macroscopic properties. Experimental results show that the compactness of material crystal structure has a significant impact on electrical properties.

## 2. Experimental Method

### 2.1. Experimental Materials and Samples

Our research group previously set the particle addition content at 3% and found that the composite material has not been effectively improved in terms of crystallization behavior and conduction current performance [30]. Relevant studies have shown that the addition concentration of particles [24] and whether the particles are treated with coupling agent [31] have a critical impact on the properties of composite materials. In order to improve the crystallization properties, conductivity current performance and space charge inhibition of micro-nano composite materials, the particle addition content was set as 1%, and MMT was also treated with coupling agent KH570 in this article. The SiO_2_ selected in the experiment was from Beijing Deke Daojin Science and Technology (Beijing, China) with a size of 30 nm. The MMT was purchased from Qinghe Chemical Plant in Zhangjiakou, China. The particle size of the original montmorillonite is about 40–70 μm, and the cation exchange capacity is 0.9–1.2 mol/kg. Silane coupling agent (KH570) was purchased from Beijing Deke Daojin Science and Technology. Low density polyethylene (LDPE) was purchased from Jinshan Petrochemical Company (Shanghai, China), with a density of 0.924 g/cm^3^ and a melting index of 2 ± 0.3 g/ (10 min 2.16 kg).

Hydrophobic SiO_2_ treated with KH570 coupling agent and MMT modified by organic intercalation of KH570 and Octadecyl Trimethyl Ammonium Chloride were melted and blended with LDPE matrix by torque rheometer. Five kinds of composite materials were prepared, both the content of SiO_2_ and MMT are 1 wt%, and the order of adding SiO_2_ and MMT particles was changed. The specific operations are as follows:

The temperature was set at 140 °C and the speed was 50 r/min by a computer connected to the internal mixer. Before the formal experiment, it is necessary to clean the internal mixer with pure LDPE, and then prepare the samples required for the experiment. Firstly, the low-density polyethylene material was added to the internal mixer for about 20 min. After it was completely melted, the filler (SiO_2_ or MMT particles) was added for melting blending. LDPE, MMT /LDPE, SiO_2_/LDPE, SiO_2_-MMT/LDPE, and MMT-SiO_2_/LDPE composite materials were prepared successively according to the above operation. During the preparation of SiO_2_-MMT /LDPE and MMT-SiO_2_/LDPE composite materials, the addition order of SiO_2_ and MMT particles was changed. SiO_2_-MMT/LDPE composite materials were first added with SiO_2_ particles and then MMT particles, while MMT-SiO_2_/LDPE composite materials were first added with MMT particles and then SiO_2_ particles. After materials were made, each material was cut into relatively small particles with scissors and placed in a dry environment for 24 h pretreatment. Then, plate vulcanization machine for the tabletting operation was used. The temperature of the plate vulcanization machine was set at 140 °C. The material was treated with step-up pressure method; that is, 0 MPa pressure for 5 min, 10 MPa pressure for 5 min, and finally under full pressure (15 MPa) for 5 min. Then, the samples were obtained by cooling under full pressure.

### 2.2. Crystallization Behavior

The crystal morphology of samples was observed with polarizing microscope (PLM, Leica DM2500, Leica Microsystems, Wetzlar, Germany). Firstly, the samples were immersed in a pre-configured mixture of KMnO_4_ and concentrated H_2_SO_4_ (etching solution) with a mass fraction of 5% for 5 h, and the solution was stirred every half an hour, so that the crystal morphology of various materials could be better observed under a polarizing microscope. After etching, the samples were taken out with tweezers and washed with plenty of deionized water. The washed samples were placed in an ultrasonic cleaner and cleaned again for about a quarter of an hour. After the moisture on the surface of the sample evaporates, each sample was placed under a polarizing microscope for observation and photographing.

The crystallization and melting process of the samples were measured by the method of differential scanning calorimetry (DSC), and DSC-1 equipment of Mettler Toledo was used. The temperature rises and falls rates were both selected to be 10 °C/min and carried out under a nitrogen atmosphere. The weighing amount of each sample is 10–15 mg. The samples were first raised from room temperature (25 °C) to 140 °C and then cooled to room temperature (25 °C). This is to eliminate the thermal history of various materials and make the polymer melt into a uniform molten state distribution. The ordered structure in the polymer melt has been eliminated, and it has become a completely disordered melt (in theory, completely eliminated). After that, the temperature was slowly raised to 140 °C again. During the heating process, the DSC curve was plotted.

### 2.3. Conduction Current

**Figure 1:**
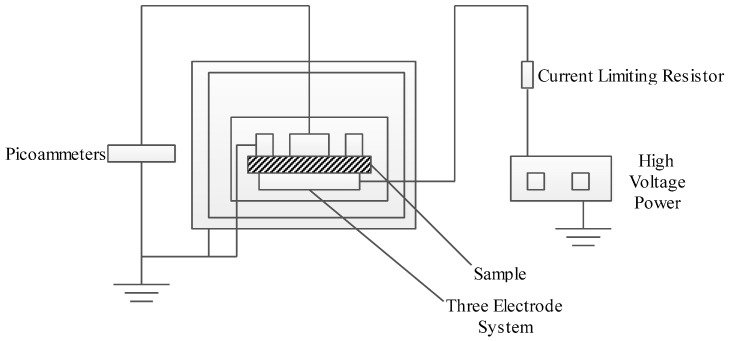
Conductance current test device.

This experiment is based on the Chinese standard GB/T 1410-2006. The measurement system consists of a high voltage DC generator, a protective resistance, a picoammeter and a three-electrode measuring unit, as shown in Figure 1. The test temperature was 25 ± 1 °C, the polarization time was 10 min, the voltage uniformly increased, the field intensity increased from 1 kV/mm to 30 kV /mm. The data required for the experiment was measured.

### 2.4. Space Charge

**Figure 2:**
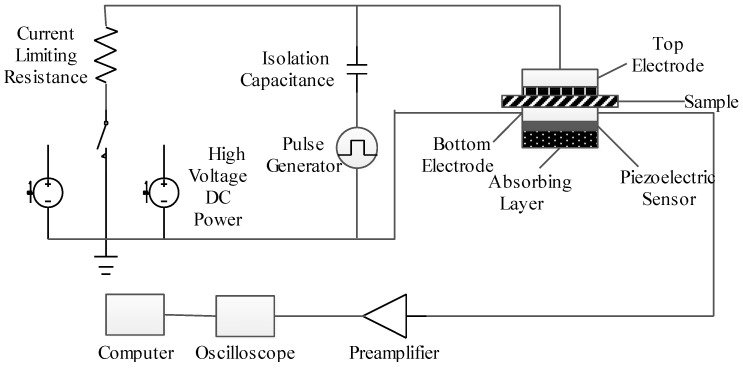
Experimental system of pulsed electro-acoustic method.

In this experiment, pulsed electro-acoustic (PEA) method is adopted. The system consists of high voltage DC power supply, pulse generator, upper and lower electrodes, preamplifier, oscilloscope and computer, etc., as shown in Figure 2. The principle is to inject the electro acoustic pulse into the interior of the test sample as a virtual probe, and the space charge will generate a force under the action of the electric pulse. Finally, the acoustic signal will be converted into an electrical signal by the piezoelectric sensor to make the external test circuit respond, so as to obtain the space charge distribution in the sample. The samples were subjected to step-by-step boosting, 10 kV/mm, 20 kV/mm and 30 kV/mm DC field intensity were applied to the sample for polarization. The space charge characteristic under the preloading field intensity was obtained by polarization for 30 min at each stage.

## 3. Results and Discussion

### 3.1. Observation of Composite Materials Crystal Morphology

**Figure 3:**
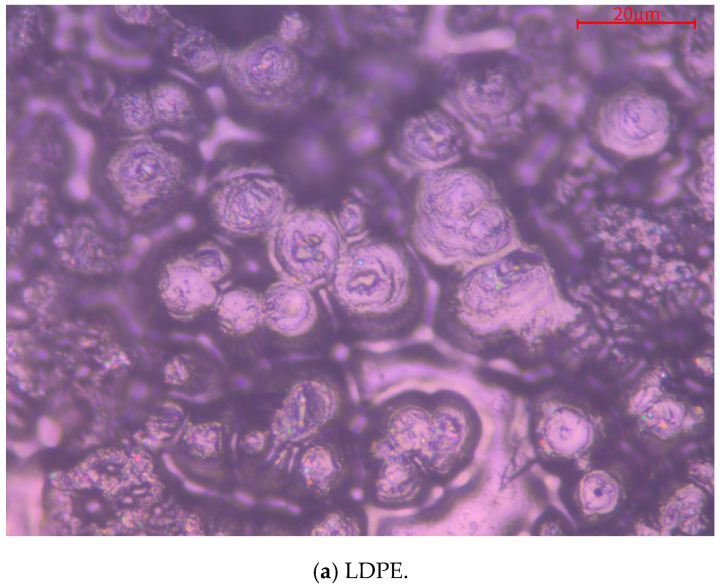

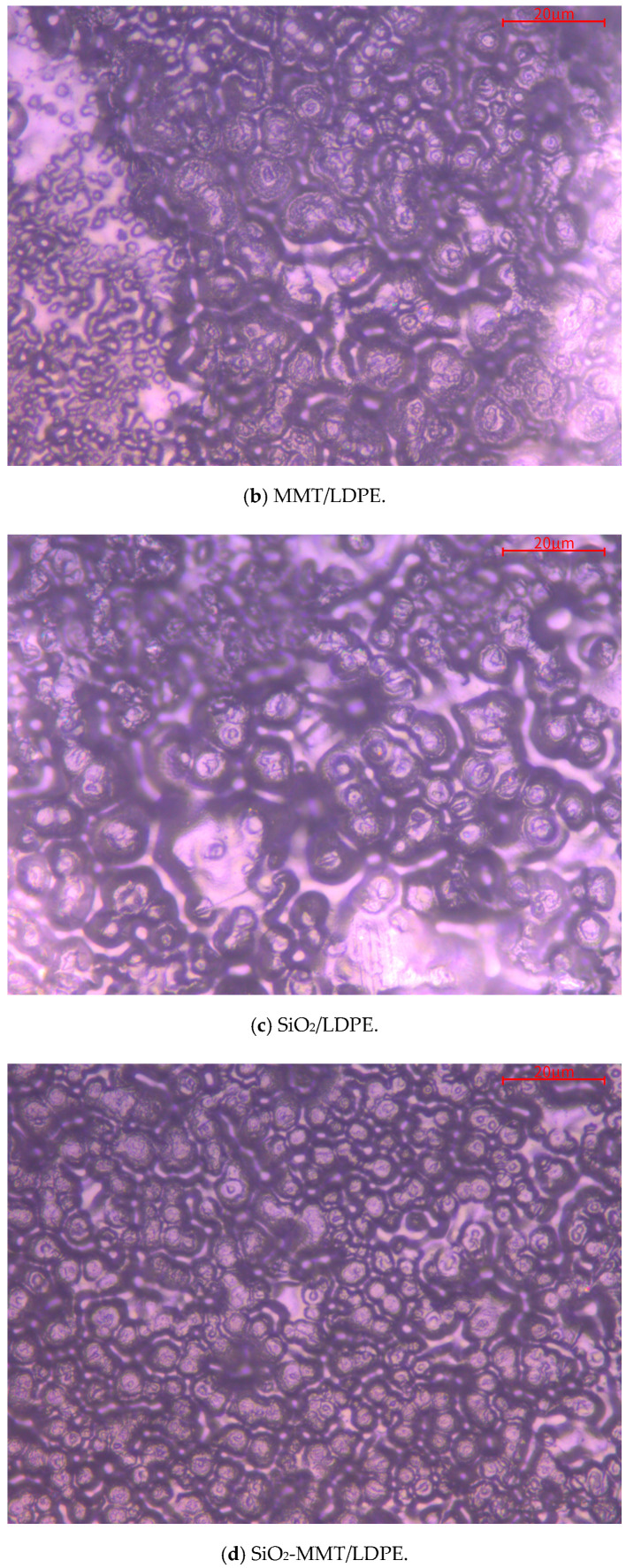

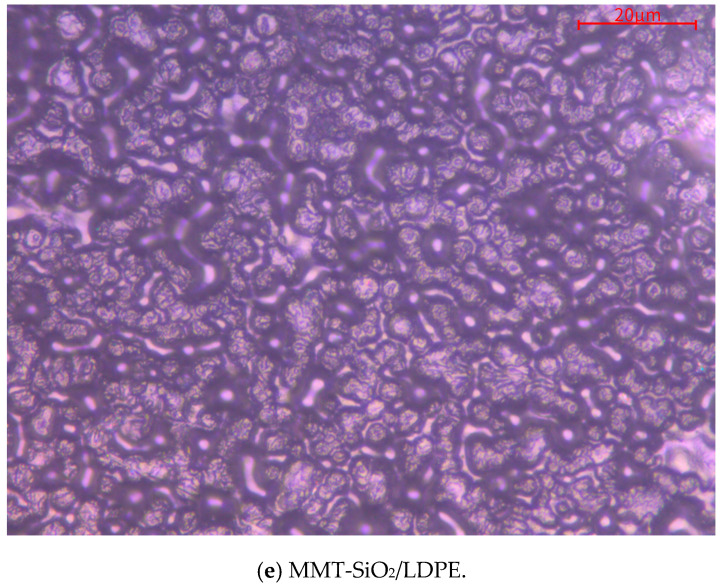
The crystalline morphology of LDPE and composites after etching observed under polarizing microscope. (**a**) PLM image of LDPE; (**b**) PLM image of MMT/LDPE; (**c**) PLM image of SiO_2_/LDPE; (**d**) PLM image of SiO_2_-MMT/LDPE; (**e**) PLM image of MMT-SiO_2_/LDPE.

The composite materials prepared by compounding SiO_2_ and MMT separately and changing the order between them and blended with LDPE. Figure 3 shows their crystal morphology observed under a polarizing microscope (PLM) after being etched with a mixed solution of concentrated sulfuric acid and potassium permanganate; the scale in the upper right corner is 20 μm. It can be seen that the crystals of the five samples are spherical in structure. Figure 3 also shows the unit cell arrangement of each composite material. If the unit cell size is large and the distance between the unit cells are long, the crystal structure of the material will be loose. The crystal structure of the material with a small unit cell size and close distance between the unit cells will be relatively tight. In Figure 3a, the unit cell size of LDPE is the largest, and the crystal structure is loose. In Figure 3b, it can be seen that after MMT is added to the matrix, the size of unit cell becomes smaller and the spacing of unit cell is reduced. Therefore, the crystal structure of MMT/LDPE is relatively tight. This may be due to the effect of coupling agent KH570, which makes the contact effect between MMT and LDPE better, thus making the internal crystal structure of the material more compact. Figure 3c shows that after the addition of SiO_2_, the unit cell size was further reduced, but the unit cell spacing was slightly larger. It can be seen from Figure 3d that the unit cell size and spacing of SiO_2_-MMT/LDPE composite prepared by adding SiO_2_ particles first and MMT particles later are further reduced. In Figure 3e, the unit cell size and spacing of the composite material MMT-SiO_2_/LDPE with MMT particles added first and then SiO_2_ particles are slightly reduced compared with SiO_2_-MMT/LDPE with SiO_2_ particles added first and then MMT particles, and the internal crystal structure of the material is more compact. This is because the large-sized particles are added first, and the large-sized particles occupy a certain space in the matrix, and then the small-sized particles are added to further fill the gaps. The large-size particles added later in the SiO_2_-MMT/LDPE composite material squeezed and destroyed the structure formed by the small-size particles, so the structure is not as tight as the MMT-SiO_2_/LDPE.

### 3.2. DSC Testing of Composite Materials

**Figure 4:**
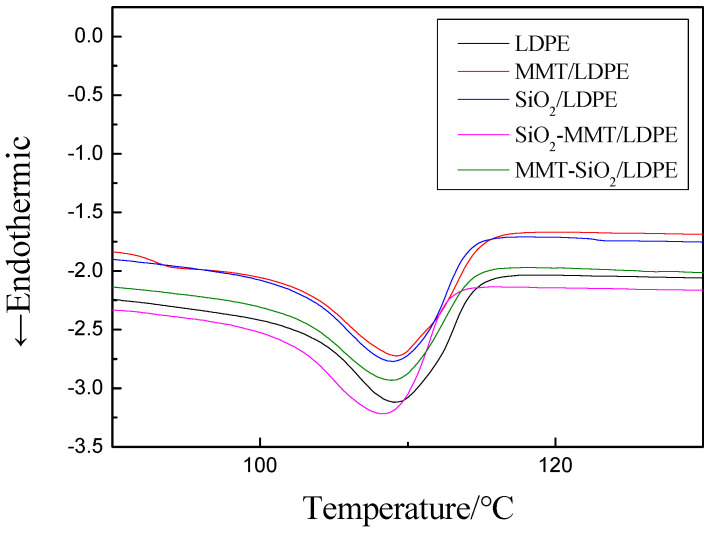
DSC curves of composite materials.

DSC-1 differential scanning calorimeter was used to test the crystallinity and melting temperature of composite materials. The measurement condition was nitrogen atmosphere, the rising and cooling rate was 10 °C/min, and the temperature range was 25 °C to 150 °C. The test results were shown in Figure 4. The melting peak temperature value is obtained directly from the instrument, as shown in Table 1.

The melting enthalpy $\Delta H_{m}$ during the melting of each composite material can be calculated by Equation (1) [32].

(1) $$H_{m}=60\int_{T_{i}}^{T_{f}}\frac{Q_{H\left(T\right)}}{B}dT$$

where, $T_{i}$ and $T_{f}$ are the starting and ending temperatures of the melting peak; $Q_{H\left(T\right)}$ is the differential heating/cooling rate (W/g); *B* is the rate of temperature rise/fall, and the calculation results are listed in Table 1.

The crystallinity of the five samples can be calculated according to Equation (2), and the calculation results are shown in Table 1 [33].

(2) $$X_{c}=\frac{\Delta H_{m}}{\Delta H_{0}}\times 100\%$$

where, $H_{0}$ is 293.6 J g^−1^ (melting enthalpy of LDPE crystallization). The melting temperature, crystallinity and melting heat of the five samples are shown in Table 1.

According to Figure 4 and Table 1, it can be seen that the crystallinity of the composite materials has been improved to different degrees. This is because after the addition of particles, the crystal area of the material is enlarged due to the heterogeneous nucleation. Among them, the crystallinity of SiO_2_/LDPE material increased significantly, which may be due to the small size of SiO_2_ particles and the positive effect of heterogeneous nucleation. SiO_2_ particles were added first, and then MMT; however, the size of MMT particles is too large—the addition of MMT particles into the matrix will change the original crystal structure. At the same time, the large-size particles added later, as a nucleation center, will form new unit cells, which will inevitably squeeze the unit cell formed by the matrix and the small-size particles added first, and the original crystal structure will be extruded and destroyed, so that the crystallinity of the composite material is not as good as that added SiO_2_ particles alone. MMT particles were added first and SiO_2_ particles were added later, the size of MMT particles was large and the size of SiO_2_ particles was small, small-size particles was not as easy to squeeze into the material as that of the large-size particles, and unit cells formation is limited. As a result, the extrusion effect was weak and the nucleation interval could not be expanded as that of the large-size particles, so the crystallinity was slightly smaller than that of SiO_2_-MMT/LDPE. At the same time, adding particles will build many new heat conduction channels in the material, so that the melting temperature of the composite is improved.

From the experimental results, the smaller the particles, the more obvious this effect. When two kinds of particles are added, the latter will introduce into the heat conduction channel and extrude the original, and the extrusion effect is slightly greater than the introduction effect, so the melting temperature is reduced. In this process, large-size particles will be easier to squeeze in, easier to form gaps, and more heat conduction channels will be formed, which makes the melting temperature of SiO_2_-MMT/LDPE slightly higher than that of MMT-SiO_2_/LDPE.

### 3.3. Conductance Current

**Figure 5:**
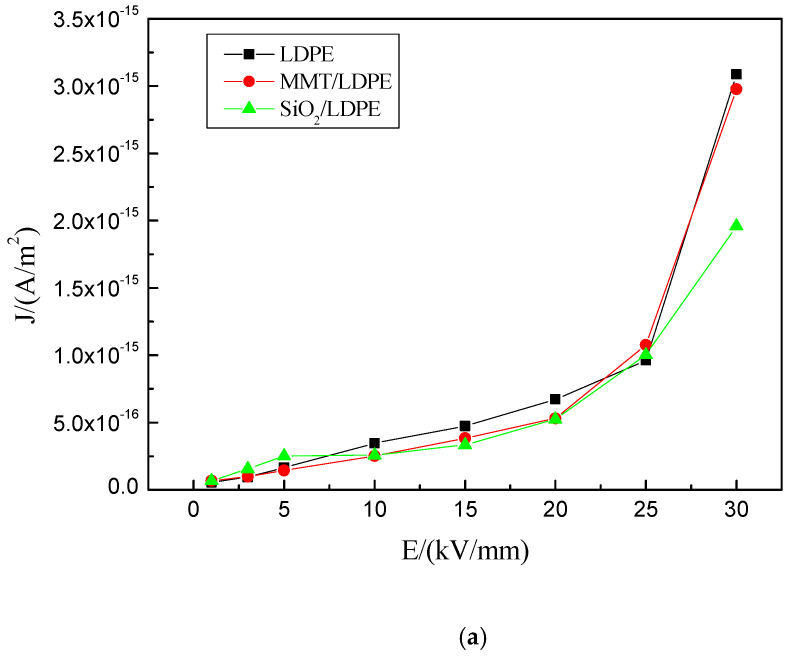

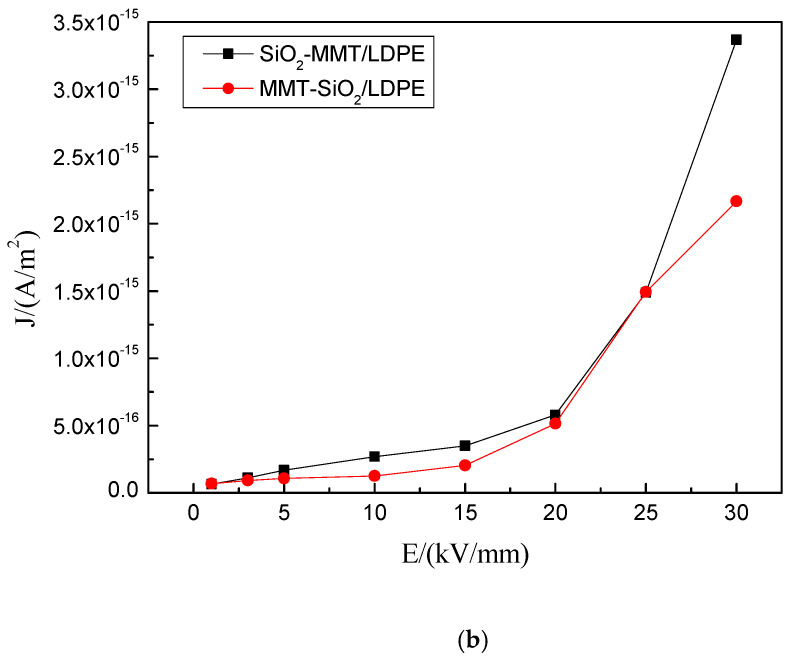
Curve of the conductance current of LDPE and composite materials changing with the field intensity. (**a**) The relationship between conductance current and field intensity of LDPE, MMT/LDPE, and SiO_2_/LDPE; (**b**) The relationship between conductance current and field intensity of SiO_2_-MMT/LDPE and MMT-SiO_2_/LDPE.

Figure 5 shows the curve of the conductance current of each sample changing with the field intensity. It can be seen from the figure that the conductance current of pure LDPE material increases linearly with the increase of the field intensity in the range before 25 kV/mm, which conforms to Ohm law. When the electric field strength is greater than 25 kV/mm, the current rises sharply, the anode starts to emit holes, and space charge accumulation will appear in the material. The current at this time is usually called the space charge limiting current, and the field intensity threshold at this time is the transition field intensity of space charge limiting current [34]. The transition field intensity is related to the trap depth inside the material, which can be described by Equation (3) [35].

(3) $$E_{\Omega }\propto exp\left[\frac{E_{t}}{KT}\right]$$

where $E_{\Omega }$ represents transition field intensity threshold, and $E_{t}$ represents trap depth. After the addition of particles, it can be found that the threshold of field intensity decreases, and the threshold field intensity of the micro-nano composite material seriously decreases, which can indicate that the trap depth of the composite material becomes shallow to some extent. Traps are mainly caused by the incompleteness of the internal structure of the medium. They are generally divided into two categories: physical traps and chemical traps. The physical traps are determined by the structure of the medium itself, and the chemical traps are related to the impurities introduced into the medium. The two are inseparable and interrelated [36]. Combined with PLM experimental data, it can be found that after adding particles, the internal structure of the material becomes more compact and tends to be complete, which will cause some deep traps to become shallow and even disappear.

According to Equation (3), it is known that the threshold of field intensity will be reduced at this time, which is consistent with the experimental results in Figure 5. It can be seen from Figure 3 and Figure 4 and Table 1 that after adding particles, the structure of the composite is compact and the crystallinity is improved, which further hinders the movement of the molecular chain, reduces the free travel of electrons, and reduces the conductance current. In the SiO_2_-MMT/LDPE composite material, small-sized particles are added first and then large-sized particles; the squeezing of large particles expands the space for the electron movement, and a new conductive channel may even be constructed. Thus, the conductance current will increase.

**Figure 6:**
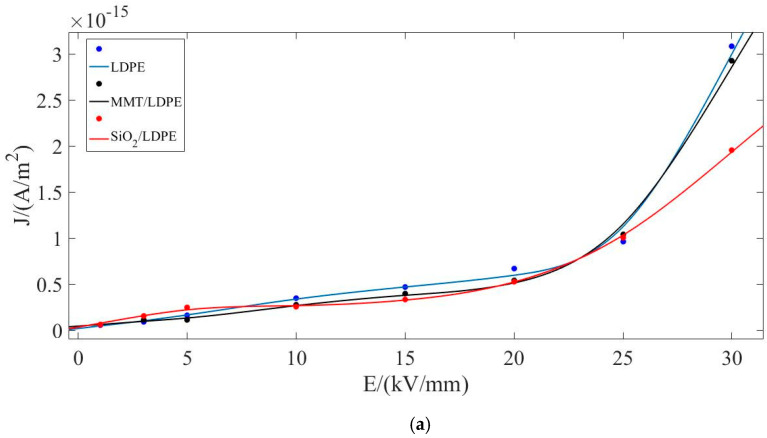

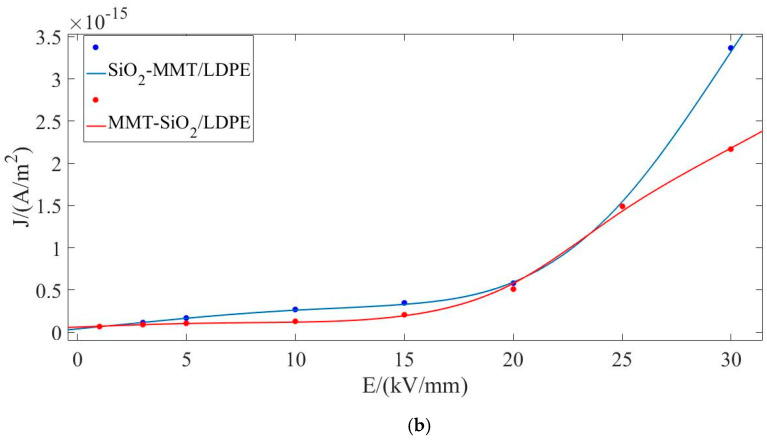
Curve-fitting figure of conductance current of each composite material changing with field intensity. (**a**) The fitting figure for LDPE, MMT/LDPE, and SiO_2_/LDPE; (**b**) The fitting figure for SiO_2_-MMT/LDPE and MMT-SiO_2_/LDPE.

In order to better analyze the data, MATLAB software was used in this paper to fit the data in Figure 5, and the results in Figure 6 were obtained [37]. According to reference [34], the current at the inflection point of the curve is space charge limited current. From Calder’s law, the space charge limiting current relation is obtained as follows:

(4) $$j=\frac{9}{8}\frac{\varepsilon \varepsilon_{0}\mu U^{2}}{d^{3}}$$

where, μ is the carrier mobility, ε is the high-frequency permittivity, $\varepsilon_{0}$ is the vacuum permittivity.

When there are traps in the medium, the traps will capture the charge, so the current rises slowly. If energy is applied to the medium all the time and the trap is filled, the current will rise sharply at this time, and the transition voltage meets the following equation:

(5) $$U=\frac{8}{9}\frac{en_{0}d^{2}}{\varepsilon }$$

where, $n_{0}$ is carrier concentration.

We can deduce the values of trap density and carrier mobility by Equations (4) and (5) [38], and the results are shown in Table 2.

According to the results in Table 2, the PLM experiment and DSC data analysis results can be further verified. The addition of particles will make the material tend to be normalized, making the original deep traps in the material become shallow or disappear, and thus the density of the traps will decrease.

Combined with the PLM experimental figure analysis, it can be seen that the closer the structure, the smaller the trap density inside the composite material, which further proves the previous inference. Comparing SiO_2_-MMT/LDPE with MMT-SiO_2_/LDPE, the former will change the local state structure during the crowding process due to the large size of the MMT particles added later, which will lead to the increase of traps, thus resulting in the result that the trap density is higher than MMT-SiO_2_ /LDPE. From the carrier mobility of the composites, it can be seen that the addition of particles will hinder the movement of molecular chain and the migration of carriers, while the addition of large-size MMT particles weakens the blocking effect and increases the carrier mobility of SiO_2_-MMT/LDPE.

### 3.4. Space Charge

**Figure 7:**
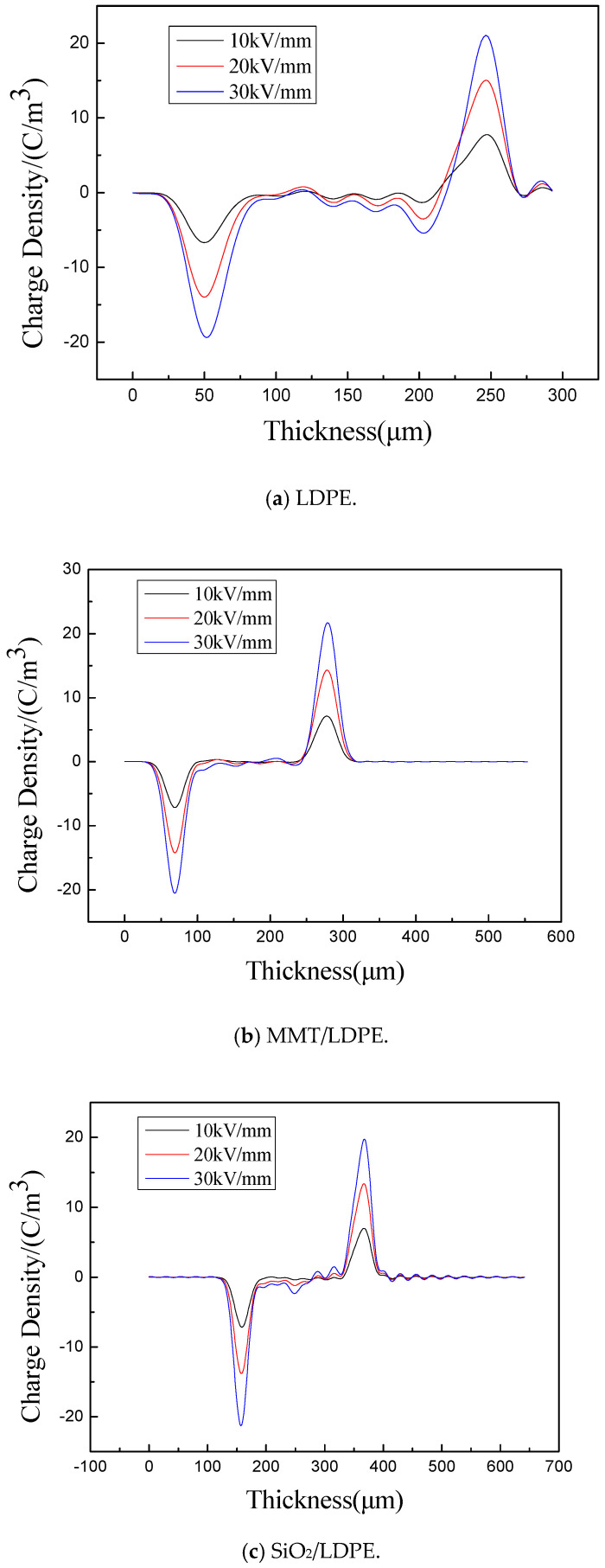

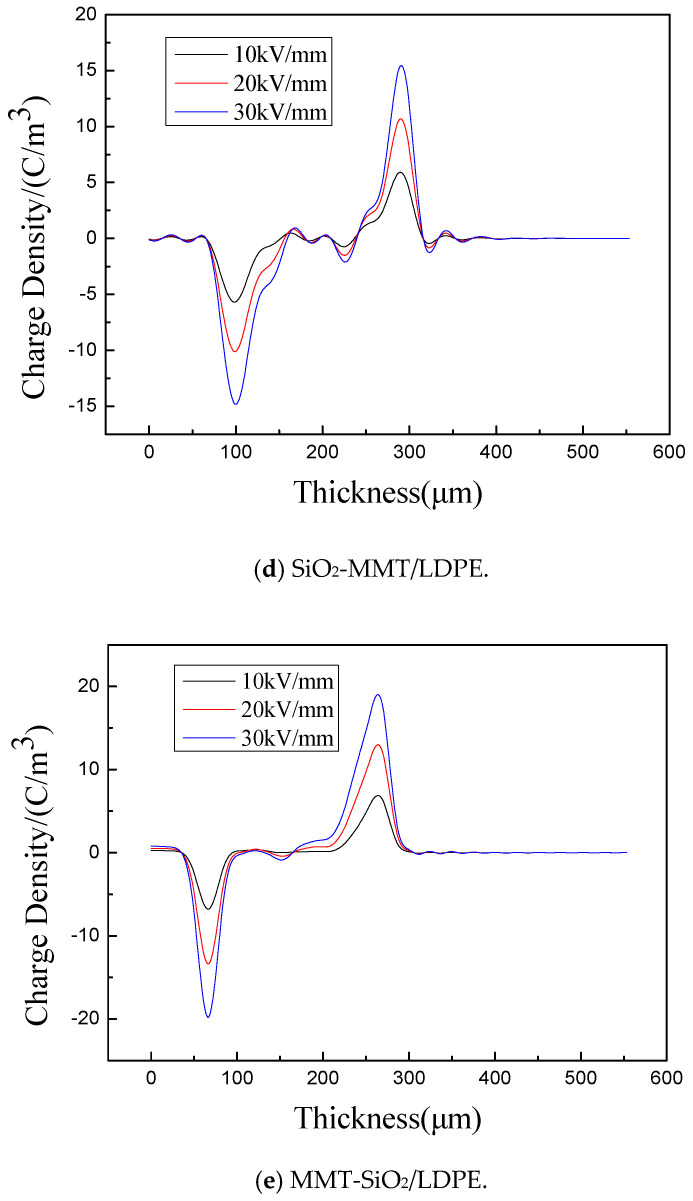
Space charge distribution of LDPE and composite materials under polarization electric field after 30 min. (**a**) Space charge distribution of LDPE; (**b**) Space charge distribution of MMT/LDPE; (**c**) Space charge distribution of SiO_2_/LDPE; (**d**) Space charge distribution of SiO_2_-MMT/LDPE; (**e**) Space charge distribution of MMT-SiO_2_/LDPE.

Figure 7 shows the cumulative distribution curve of space charge for each sample. The ordinate is the space charge density, and the abscissa is the space distance. When an electric field is applied, the carrier injected into the electrode will form a homopolar charge near the electrode due to the trapping effect in the material, thus reducing the field intensity nearby the electrode. When the heteropolar charge migrates against the direction of electric field, it changes the space charge distribution in the medium by trapping, decaying or compounding with the same polarity charge [39]. It can be seen from Figure 7a that a small amount of negative charge accumulates in the middle of LDPE sample with field strength of 10 kV/mm. With the increase of field strength, the amount of charge accumulation also increases. This is because the molecular structure and morphology of polyethylene are related to carrier injection, transport and trap. Polyethylene consists of a crystalline and amorphous phase, residual free volume, the double bond, end group, crystal phase and amorphous phase interface, leading to the increase of the local state, the local state can be used as carrier trap, capture and hinder the carrier migration, formation of space charge. When the field strength increases, the impurities in LDPE will be ionized, and a large number of anions and cations will be produced. Their moving rate is relatively slow and they are more easily trapped, so a large amount of space charge will be accumulated before reaching the electrode.

After adding MMT particles, the space charge in the sample dissipates obviously. It can be seen from the PLM figure that the addition of MMT particles makes LDPE crystal perfect and structure more tightly regular, eliminating some LDPE structural defects, making some traps become shallow or disappear, which makes it difficult to capture charge and reduces the accumulation of space charge near the electrode. The results in Table 2 show that after adding the particles, the carrier migration is hindered. According to the DSC experimental results, the crystallinity is greatly improved. In addition, after the addition of MMT particles, a large number of interfaces are introduced. The interfaces between the crystalline and amorphous regions and between particles play a scattering role on carriers, further slowing down the carrier migration, which is greatly conducive to the neutralization of positive and negative charges. Under multiple actions, the space charge inhibition effect of MMT/LDPE is obvious, as shown in Figure 7b.

PLM results show that the introduction of SiO_2_ particles can also improve the structure of the composite, resulting in the reduction of space charge accumulation of SiO_2_/LDPE composite, but also lead to the introduction of deep traps [40]. As shown in Figure 7c, after the charge is captured by the deep trap, the homopolar charge accumulation will be formed, which is especially prominent under a high electric field [41]. In this way, an anti-electric field at the interface between the electrode and the sample will be formed and inhibit the further injection of electrons or holes. Due to the suppression effect of particles and the scattering effect of a large number of interfaces inside the medium, the space charge accumulation is reduced. Comparing Table 2 and Figure 7, the internal trap density of MMT/LDPE is 18.3% higher than that of SiO_2_/LDPE, while the space charge accumulation of the material is significantly lower than that of the latter, which indicates that most of the traps in MMT/LDPE are shallow traps, while those in the latter are deep traps. According to reference [42], shallow traps capture carriers and provide transition channels for carrier transport. Electrons continuously jump between the conduction band and shallow traps to complete the migration process. As a result, the carrier mobility of MMT/LDPE in Table 2 is 3.48% higher than that of SiO_2_/LDPE.

The first step is to add SiO_2_ particles and then MMT particles to create the positive and negative charges inside the material interlace. This occurs because of the addition of larger particles creates gaps in tightly packed structures. It can be seen from Table 2 that this weakens the effect of suppressing carrier migration and cannot neutralize positive and negative charges. Moreover, these gaps lead to the generation of new localized states in the material, which results in a large amount of space charge accumulation in the medium, resulting in the result in Figure 7d.

However, adding MMT particles first and then the SiO_2_ particles makes the material structure compact and reduces the number of traps. Due to the introduction of MMT and SiO_2_ particles, the effect of deep traps and interfaces in the medium is enhanced, and the anti-electric field and interface scattering factors are more intense. Therefore, the space charge suppression effect of the MMT-SiO_2_/LDPE composite material is significantly improved. The experimental results are shown in Figure 7e.

## 4. Conclusions

From the PLM experimental results, it can be confirmed that the introduction of micron MMT particles and nano-SiO_2_ particles into the matrix LDPE will make the internal crystal structure of the material more compact. The structure of the first large and then small addition mode (adding MMT particles first and then SiO_2_ particles) is more compact than that of the small first and then large addition mode (adding SiO_2_ particles first and then MMT particles). The latter will have a slight increase in structure gap and larger nucleation space due to the crowding of large particles. From the DSC experimental results, the simultaneous addition of micron MMT particles and nano-SiO_2_ particles will destroy the original crystal structure. In addition, the crystallinity of SiO_2_-MMT/LDPE and MMT-SiO_2_/LDPE is slightly lower than that of a single particle.

From the curve of conductance current changing with field intensity and with the addition of particles, the structure of the material becomes compact. Some traps become shallow or disappear, and the threshold value of field strength is advanced. Meanwhile, the compact structure can effectively limit the movement of the molecular chain and reduce the conductance current. The MMT-SiO_2_/LDPE composite prepared by adding large-size MMT particles and then adding small-size SiO_2_ particles has an obvious blocking effect on carrier migration.

From the results of the space charge accumulation distribution curve, the compact and complete structure will make part of the localized state in the material become less than matrix, and cause the traps inside the material to be shallower or even disappear. After particles are added, the multiple effects of scattering and the counter electric field will increase the inhibition effect of the composite material on the space charge. The SiO_2_-MMT/LDPE composite material was made by adding small-sized SiO_2_ particles first and then large-sized MMT particles. Due to the later squeezing of large particles (MMT), there are gaps in the structure, which reduces the effect of suppressing carrier migration. At the same time, new localized states appear in the material, which results in new traps and obvious space charge accumulation. The structure of MMT-SiO_2_/LDPE composite material made by adding large-size MMT particles first and then small-size SiO_2_ particles is more compact. While fully hindering the migration of carriers, it also aggravates the effects of scattering and anti-electric field and has a significant suppression effect on space charges.

## Figures and Tables

**Table 1:** Melting peaks and crystallinities of all samples.

Samples	Melting Peak Temperature Tm/℃	Crystallinity Xc/%	Melting Heat /J·g^−1^
LDPE	108	30.89	90.69
MMT/LDPE	108.18	33.73	99.03
SiO_2_/LDPE	108.01	35.33	103.72
SiO_2_-MMT/LDPE	107.77	32.39	95.10
MMT-SiO_2_/LDPE	107.49	31.59	92.75

**Table 2:** Calculation results of trap density and carrier mobility of each sample.

Samples	d (μm)	U (kV)	n_t_ (m^3^)	μ_e_ (m^2^/V^−s^)
LDPE	210	4.65	1.59 × 10^19^	1.25 × 10^−23^
MMT/LDPE	210	4.38	1.42 × 10^19^	1.19 × 10^−23^
SiO_2_/LDPE	210	3.30	1.20 × 10^19^	1.15 × 10^−23^
SiO_2_-MMT/LDPE	210	3.60	1.17 × 10^19^	1.24 × 10^−23^
MMT-SiO_2_/LDPE	210	3.21	1.04 × 10^19^	8.07 × 10^−24^
